# Two structurally discrete GH7-cellobiohydrolases compete for the same cellulosic substrate fiber

**DOI:** 10.1186/1754-6834-5-21

**Published:** 2012-04-11

**Authors:** Fernando Segato, André R L Damasio, Thiago Augusto Gonçalves, Mario T Murakami, Fabio M Squina, MariadeLourdesTM Polizeli, Andrew J Mort, Rolf A Prade

**Affiliations:** 1 Department of Microbiology & Molecular Genetics, Oklahoma State University, Stillwater, OK, USA; 2 Department of Biochemistry and Molecular Biology, Oklahoma State University, Stillwater, OK, USA; 3 Laboratório Nacional de Biociências (LNBio), Campinas, Sao Paulo, Brazil; 4 Laboratório Nacional de Ciência e Tecnologia do Bioetanol (CTBE), Centro Nacional de Pesquisas em Energia e Materiais, Campinas, Sao Paulo, Brazil; 5 Department of Biochemistry, Ribeirão Preto School of Medicine, Ribeirão Preto, Sao Paulo, Brazil; 6 Biology Department, FFCLRP, Universidade de São Paulo, Ribeirão Preto, Sao Paulo, Brazil

**Keywords:** Cellobiohydrolase, Cellobiohydrolase I, Cellobiohydrolase D, *Aspergillus niveus*, *Aspergillus fumigatus*, Crystalline cellulose breakdown, Biofuels, Cellulases, Biomass decomposition

## Abstract

**Background:**

Cellulose consisting of arrays of linear beta-1,4 linked glucans, is the most abundant carbon-containing polymer present in biomass. Recalcitrance of crystalline cellulose towards enzymatic degradation is widely reported and is the result of intra- and inter-molecular hydrogen bonds within and among the linear glucans. Cellobiohydrolases are enzymes that attack crystalline cellulose. Here we report on two forms of glycosyl hydrolase family 7 cellobiohydrolases common to all *Aspergillii* that attack Avicel, cotton cellulose and other forms of crystalline cellulose.

**Results:**

Cellobiohydrolases Cbh1 and CelD have similar catalytic domains but only Cbh1 contains a carbohydrate-binding domain (CBD) that binds to cellulose. Structural superpositioning of Cbh1 and CelD on the *Talaromyces emersonii* Cel7A 3-dimensional structure, identifies the typical tunnel-like catalytic active site while Cbh1 shows an additional loop that partially obstructs the substrate-fitting channel. CelD does not have a CBD and shows a four amino acid residue deletion on the tunnel-obstructing loop providing a continuous opening in the absence of a CBD. Cbh1 and CelD are catalytically functional and while specific activity against Avicel is 7.7 and 0.5 U.mg prot^-1^, respectively specific activity on *p*NPC is virtually identical. Cbh1 is slightly more stable to thermal inactivation compared to CelD and is much less sensitive to glucose inhibition suggesting that an open tunnel configuration, or absence of a CBD, alters the way the catalytic domain interacts with the substrate. Cbh1 and CelD enzyme mixtures on crystalline cellulosic substrates show a strong combinatorial effort response for mixtures where Cbh1 is present in 2:1 or 4:1 molar excess. When CelD was overrepresented the combinatorial effort could only be partially overcome. CelD appears to bind and hydrolyze only loose cellulosic chains while Cbh1 is capable of opening new cellulosic substrate molecules away from the cellulosic fiber.

**Conclusion:**

Cellobiohydrolases both with and without a CBD occur in most fungal genomes where both enzymes are secreted, and likely participate in cellulose degradation. The fact that only Cbh1 binds to the substrate and in combination with CelD exhibits strong synergy only when Cbh1 is present in excess, suggests that Cbh1 unties enough chains from cellulose fibers, thus enabling processive access of CelD.

## Background

Biofuel generation from sources such as cornstarch, sugarcane or sweet sorghum syrups, produces large amounts of biomass waste products. For example, over 90% of the plant is unused in the case of ethanol production from cornstarch [1-3]. Current commercial enterprises produce ethanol from cornstarch, sugar cane or sweet sorghum syrups and large amounts of waste biomass accumulate alongside refineries and most of them are not recycled. Biofuels production would therefore be significantly more efficiently utilized if this biomass could be converted into fermentable sugars.

Biomass polysaccharides consist of cellulose, hemicellulose and pectin. Cellulose is a linear, crystalline self-assembled nanofiber formed from the linear polymer containing exclusively glucose monomers linked through β-1,4-glycosydic bonds [1-4]. Recalcitrance of cellulose towards enzymatic degradation is a widely reported phenomenon [1,5,6] and is the result of the wide range of possible intra- and inter-molecular hydrogen bonds within and among linear cellulose molecules assuming crystalline or amorphous cellulosic nanofiber structures. Cell walls from different plants contain various amounts of crystalline/amorphous cellulose fibers [7], reflected by the relative crystalinity index (RCI).

Because cellulose is structurally complex and crystalline in nature, it is recalcitrant towards microbial or enzymatic attack. Unfortunately, current pretreatment methods – e.g., acid hydrolysis [8,9] or pyrolysis [10,11], generate compounds that inhibit subsequent fermentation. Enzymatic hydrolysis of cellulose results in glucose the universal carbon source for all organisms to drive oxidative metabolism including the production of biofuels, chemicals and pharmaceuticals.

A complete enzymatic cellulose degrading system consists of at least three related partially redundant biochemical reactions. Endo-glucanases (EC 3.2.1.4) randomly hydrolyze internal glycosydic bonds to decrease the length of the cellulose chain and multiply polymer ends [12-14]. Exo-glucanases (EC 3.2.1.91) split-off cellobiose from cellulose termini [15-17] and β-glucosidases (EC 3.2.2.21) hydrolyze cellobiose and oligomers to render glucose [18-20]. All three types of enzymes have similar catalytic domains all splitting the β-1,4-glycosidc bond between glucose molecules, however they differ in their binding and substrate interaction domains resulting in cooperation and synergism in releasing glucose [21-23].

Exo-glucanases hydrolyze cellulose chains by removing cellobiose either from the non-reducing end (GH6, EC 3.2.1.91) or reducing end (GH7, EC 3.2.1.176), which in both cases results in the release of reducing sugars (cellobiose) but little polymer length reduction [24]. In fungi, exo-glucanases are commonly known as 1,4-β-D-glucan cellobiohydrolases [25]. The main characteristic of a cellobiohydrolase is its processive action on individual cellulose chains by reiterated release of cellobiose [24,26-32].

The catalytic domain (CD) of a typical cellobiohydrolase forms a channel-shaped cavity topped by several flexible loops resulting in a tunnel like structure [33-39], which is frequently connected through a linker to a carbohydrate binding domain (CBD) [40,41]. CBDs are thought to bind to the crystalline cellulosic fiber sliding down the cellulosic surface [6,42] and carrying the CD with it. Three tyrosine residues on the CBD hydrophobic surface align with the cellulose chain adjacent to the reducing end and a fourth tyrosine residue moves from its internal position to form van der Waals interactions with the cellulose surface resulting in an induced change near the surface allowing the CBD to progress [16,40].

From a comprehensive bioinformatics survey of seven *Aspergillii* completely sequenced genomes, we found between three and five genes encoding cellobiohydrolases, typically two in each of the CAZy families GH6 and GH7, respectively. Remarkably, for each GH family 6 or 7 fungal cellobiohydrolase examined, one enzyme was linked to a CBD and a second was not.

Here we describe two GH7-cellobiohydrolases, Cbh1 and CelD from *Aspergillus niveus*. The structural differences between these two enzymes were the lack of a CBD in CelD and a conserved loop that obstructed the catalytic tunnel in enzymes that were linked to a CBD, but missing in enzymes that did not link to a CBD, ensuring that the tunnel was always open. These structural differences reflected directly on kinetic properties, thermostability, inhibition by glucose and cellobiose and the way these proteins interacted with crystalline substrate molecules. We hypothesize that the cellobiohydrolase linked to a CBD is competent to release cellulosic chains from the crystalline nanofiber and the cellobiohydrolase without a CBD can only use loose ends, without the aid of a CBD.

## Results

### *Aspergillii* hold precisely two GH7-cellobiohydrolases, one with and another without a cellulose-binding domain

Fungi and bacteria produce enzymes that at least partially degrade plant cell wall polysaccharides [43,44]. More often than not, cellulase-encoding genes appear as multiple copies in the genome of sequenced microorganisms and enzymes occur as functionally redundant with overlapping biochemical functions.

Cellobiohydrolases belonging to CAZy family GH7, attack crystalline forms of cellulose from the reducing ends of cellulose chains generating cellobiose [13,21,24,27]. Table 1 shows that the genome of almost every *Aspergillus* precisely encodes two GH7-cellobiohydrolases, one with, and another without a CBD. The one exception is *Aspergillus flavus*/*A. oryzae* (both genomes contain an almost identical nucleotide sequence [45]), which encode two GH7-cellobiohydrolase, both with no CBD. However, it is not clear if another gene with a CBD exists or the CBD domain has not yet been annotated. Other fungi such as *Neurospora crassa* OR74, *Penicillium chrysogenum* W54-1255 and *Phanerochaete chrysosporium* have a two-set of GH7-cellobiohydrolases, while *Hypocrea jecorinaHypocrea rufaHypocrea virens* and other *Trichodermales* appear to contain only one GH7-cellobiohydrolase, all with a CBD.

**Table 1:** Genome wide distribution of gh7-cellobiohydrolases

**Aspergillii**	**gh7-cellobiohydrolases**	
	**no CBM**	**CBM**
	**Prot ID Locus tag**	**Prot ID Locus tag**
*A. clavatus* NRRL1	XP_001272622 ACLA_088870	XP_001272622 ACLA_085260
*A. fumigatus* Af293	XP_750600 AFUA 6G07070	XP_751044 AFUA 6G11610
*A. nidulans* FGSC A4	XP_662780 AN5176	XP_658098 AN0494
*A. niger* CBS 513.88	XP_001392008 ANI_1_2134064	XP_001389576 ANI_1_1574014
*A. terreus* NIH 2624	XP_001212905 ATEG_03727	XP_001214180 ATEG_05002
*A. oryzae* RIB40	XP_001818879 AOR_1_608164	none
	XP_001727881 AOR_1_1654194	none
*A. flavus* NRRL-3357	XP_002380314 AFLA_067550	none
	XP_002376207 AFLA_021870	none
*P. chrysogenum* W54-1255	CAP94773 Pc18g05490	CAP85526 Pc20g01970
*Neurospora crassa* OR 74A	XP_957090 NCU05104	XP_962498 NCU07340
*Phanerochaete chrysosporium*	CAA38274 Pccbh-1	CAA38275 Pccbh 1-2
*Hypocrea jecorina*	none	GUX_1TRIRE cbn1
*Trichoderma viride* (Hypocrea rufa)	none	BAA36215 cbh1
*Hypocrea virens*	none	ACF93800 cbh1

*A. niveus* a filamentous fungus analogous to *A. fumigatus* (the genomic DNA sequence is up to 97% identical) grew at high temperatures, up to 50°C, on medium containing complex carbon sources such as cellulose and hemicellulose. As expected, the genome of *A. niveus* encoded two GH7-cellobiohydrolases: Cbh1-like 1,4-beta-D-glucan cellobiohydrolase with a linker and CBD and a CelD-like 1,4-beta-D-glucan cellobiohydrolase with no linker and CBD. Genes, *cbh1* and *celD* were expressed and proteins secreted into the medium when the fungus was grown on cellulose (e.g., Avicel) as the carbon source (data not shown).

**Figure 1:**
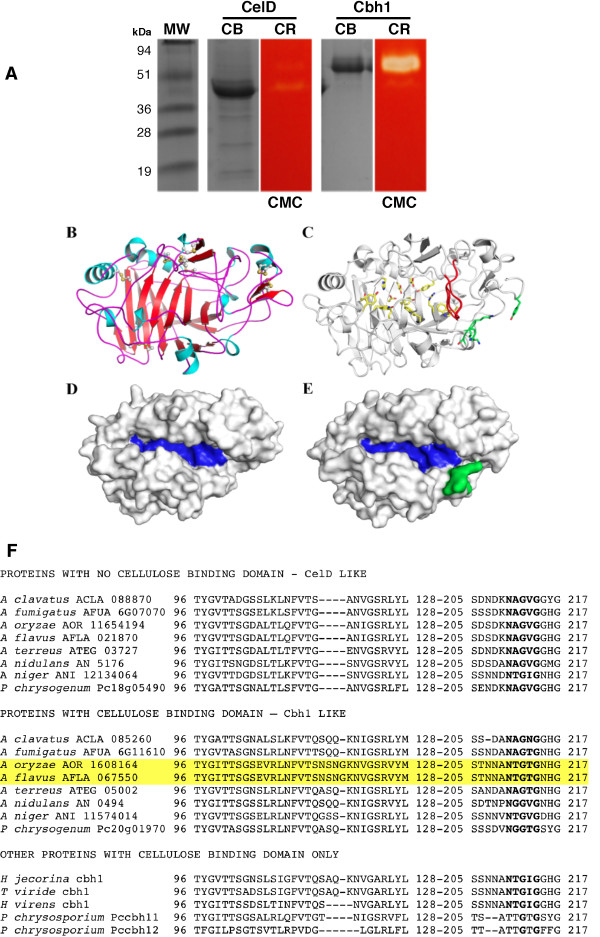
**Structural overview of Cbh1 and CelD catalytic homologs active on cellulosic substrates.** Panel **A** SDS–PAGE comassie-blue (CB) protein and carboxymethylcellulose congo-red (CR) enzyme activity staining of secreted Cbh1 and CelD proteins recovered from two-day shake-flask cultures. Cbh1 showed a strong activity against CMC while CelD showed lesser activity on CMC. Restraint-based modeling of Cbh1 and CelD identified a flexible catalytic loop and a lid-like loop obstructing the substrate tunnel. Panel **B**, cartoon representation of CelD, colored by secondary structure elements. Disulphide bonds are shown in ball-and-stick representation. Panel **C**, CelD/Cbh1 catalytic domain structural overlay. The active-cleft residues are shown as stick carbon atoms in yellow. The flexible catalytic loop, common to all GH7 cellobiohydrolases is shown in red and the three-residue lid-type insertion within the 94–103 loops only present in Cbh1 is drawn as sticks with carbon atoms in green. CelD (Panel **D**) and Cbh1 (Panel **E**) surface model prediction highlighting the catalytic substrate tunnel in blue and the obstructing lid-type loop in green. For a clearer view of the substrate channel, some residues at the protein surface were omitted. Alignment of the 94–103 lid-like and flexible catalytic loops of GH7-cellobiohydrolases (Panel **F**). All CelD like proteins with no CBD show a four amino acid deletion that opens the catalytic tunnel (shown in **D** and **E**).

Both *cbh1* and *celD* genes have been expressed as client proteins in *A. nidulans*, under the control of the maltose promoter, translated and secreted via the glucoamylase signal peptide [46] where they exhibited “in situ” activity towards carboxymethylcellulose (Figure 1A). Both proteins were purified by ion exchange followed by gel filtration chromatography and used for comprehensive biochemical analysis (See Methods and Additional file 1: Table S 1).

The Cbh1 and CelD catalytic domains share 72% identity and 82% similarity in their amino acid sequences. The main difference between these proteins is the presence of a cellulose-binding domain in Cbh1 and the absence of such a domain in CelD. The objective of this study was to investigate the mechanistic differences between these two enzymes.

### Minor structural differences in Cbh1 and CelD are catalytically relevant

Cbh1 and CelD catalytic domains (CD) were modeled based on the 3D-structural template derived from *Talaromyces emersonii* cellobiohydrolase 1 (PDBID: 1Q9H, [47]). Modeled structures were subjected to energy minimization and final models presented high-quality local and overall stereochemistry with approximately 98% of amino acid residues lying in allowed regions of the Ramachandran plot (Figure 1B-E).

The Cbh1 and CelD catalytic core comprises a typical CaZy GH7 family β-sandwich surrounded by numerous surface loops, which outline the substrate-binding channel (Figure 1B-E). The native *T. emersonii* structure presented a disordered loop encompassing the amino-acid segment 190–200. This sequence was fully conserved among all other Cbh1 proteins (Figure 1F), and likely became structured upon substrate binding similarly, to what has been reported for other glycosyl hydrolases [48]. Structural superposition resulted in a RMSD value of 0.15 Cα for Cα atoms indicating high conservation of Cbh1 and CelD three-dimensional structures. The catalytically relevant residues and the substrate channel are fully conserved including Trp38, Tyr168, Asp170, Glu209, Asp211, Glu214, Trp371 and Trp380 (Figure 1C). The only significant difference was at the entrance of the catalytic tunnel formed by loops 43–63, 94–103 and 190–200 that partially blocked the catalytic channel in Cbh1, due to a four-residue insertion in the 94–103 loop (Figure 1C and 1F). In all CelD proteins, this four amino acid insertion is missing (Figure 1F) and CelD proteins do not have a CBD thus suggesting that in the absence of a CBD, the catalytic domain only functions if the substrate channel is open.

### Cbh1 and CelD are catalytically functional

Purified Cbh1 and CelD were incubated in the presence of increasing amounts of two crystalline cellulosic substrates, Avicel and cotton cellulose and the specific velocity was determined after a 120 min reaction period (For the Michaelis Menten plot refer to Additional file 2: Figure S 1). Table 2 shows both, Cbh1 and CelD responding to increasing amounts of crystalline cellulose substrates, the number of substrate molecules converted, k_cat_, over time (minutes) significantly diverged between Cbh1 and CelD; while Cbh1 turned over 22.20 and 18.45 Avicel or cotton cellulose molecules, respectively, CelD turned over only 2.62 and 3.05 molecules respectively. Thus, Cbh1 was about 6 to 8 fold more efficient in hydrolyzing crystalline cellulosic substrates. This difference was also reflected in the determined catalytic efficiency K_cat_/K_m_, which hovers from 1.22 to 0.98 and 0.06 to 0.18 depending on the substrate, respectively (see Table 2). Specific activity for both proteins reflected the catalytic differences as well as the substrate affinities for Avicel and cotton cellulose except when assayed with *p*-nitrophenyl-β-D-cellobioside (*p*NPC) where specific activity was identical, suggesting that Cbh1 and CelD catalytic domains function equally. However they differ in the way they interact with insoluble polymeric substrates.

**Table 2:** Cbh1 and CelD kinetic properties on crystalline cellulosic substrates

**Kinetic parameter**	**Avicel**		**Cotton cellulose**		**pNPC**	
	**Cbh1**	**CelD**	**Cbh1**	**CelD**	**Cbh1**	**CelD**
V_max_(μmol/min)	24.81±0.75	3.39±0.35	20.62±0.62	3.95±0.06	-	-
K_m_(mg/mL)	18.27±1.97	43.50±6.39	18.83±1.99	16.60±0.97	-	-
K_cat_(min^-1^)	22.20	2.62	18.45	3.05	-	-
K_cat_/K_m_	1.22	0.06	0.98	0.18	-	-
U mg prot^-1*^	7.66±1.21	0.49±0.04	4.57±0.02	1.19±0.06	26.20±0.20	27.31±1.40

^*-^Specific activity (U.mg prot^-1^) determined with 10 mg/ml avicel cotton cellulose or 18 mM *p*NPC at 40 C FOR 120 mins.

### Only Cbh1 binds to cellulose

**Figure 2:**
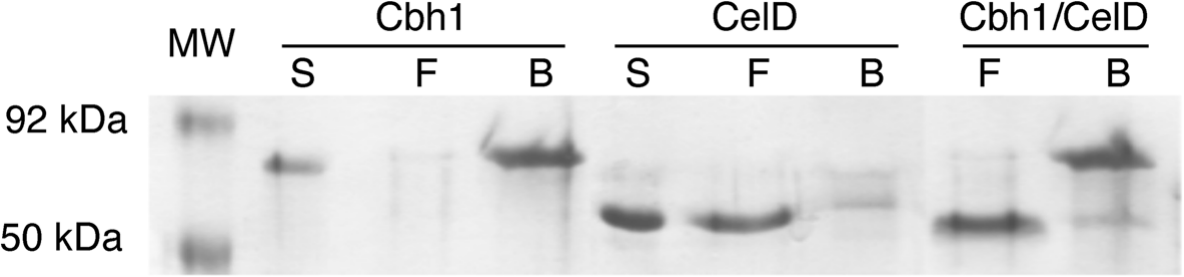
**Only Cbh1 binds to cellulose**. 20 μg of Cbh1, CelD or both (Cbh1/CelD) proteins (**S**) were incubated with 30% Avicel for 30 min, the slurry centrifuged, supernatants (**F**, free enzyme fraction) collected and enzymes removed from the pellet (**B**, cellulose-bound proteins) with 1% SDS and β-mercaptoethanol, concentrated and analyzed by SDS-PAGE.

Because CelD does not have a CBD and is biochemically active, we hypothesized that CelD could associate with Cbh1 in solution and use the Cbh1 CBD to slide along cellulose chains. However, our experiment (Figure 2) shows that CelD does not bind to cellulose alone or in combination with Cbh1, thus ruling out an association between these two enzymes in natural substrate interactions.

### Thermal inactivation leads to differential protein unfolding

**Figure 3:**
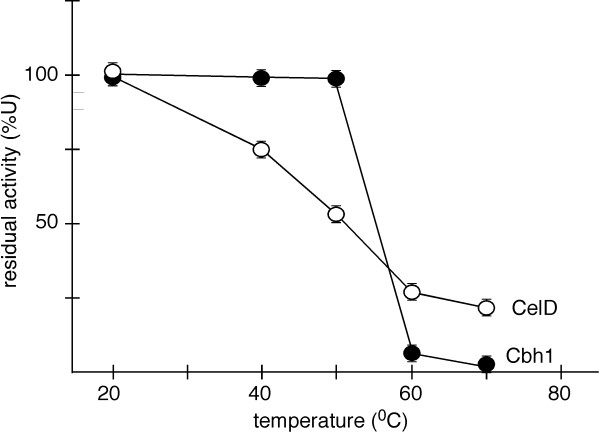
**Cbh1 and CelD differential thermal inactivation**. Cbh1 and CelD were incubated in 50 mM ammonium acetate buffer pH 5.0 in the absence of substrate for 40 min at the indicated temperature and residual activity (U) measured. Cbh1 with a CBD domain shows enhanced thermo protection in relation to CelD, which only has a catalytic domain. Time dependent inactivation curves are shown in Additional file 3: Figure S 3.

To study thermal inactivation, Cbh1 and CelD were incubated at various temperatures in the absence of substrate in 50 mM ammonium acetate buffer for 40 min and residual activity was determined with *p*NPC. Figure 3 shows that Cbh1 was slightly more stable to thermal inactivation when compared to the inactivation of CelD, suggesting that the presence of a CBD, only present in Cbh1, had a thermo protection effect similar to that observed for other proteins with CBD’s [49,50].

### Differential glucose inhibition implies two differentially adapted catalytic domains

**Figure 4:**
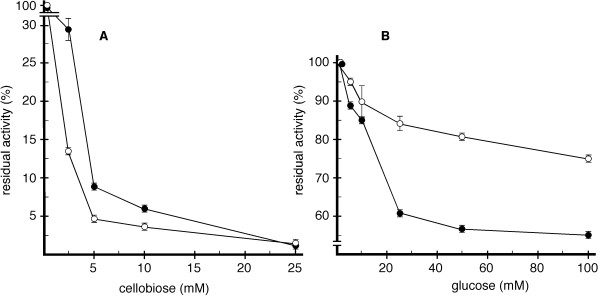
**Inhibition of Cbh1 and CelD by cellobiose and glucose**. Enzyme activity was determined with *p*NPC and increasing amounts of cellobiose (Panel **A**) or glucose (Panel **B**) added to Cbh1 (open symbols) or CelD (closed symbols) containing reactions. Both enzymes were inhibited by cellobiose and glucose, however Cbh1 was differentially (more resistant to) inhibited by glucose.

Catalytic inhibition studies of Cbh1 and CelD by cellobiose and glucose, were carried out with purified enzymes and *p*NPC as a substrate (Figure 4). In the presence of 5 mM cellobiose, 90% of the activity for both enzymes was inhibited. Addition of glucose however, had a differential effect: with 50 mM of glucose, Cbh1 retained 75% of its initial activity while CelD retained only 55%. In the presence of 100 mM of glucose, Cbh1 retained 80% of its initial activity and CelD had lost more than 50%. Thus, the rate of *p-*nitrophenyl formation from *p*NPC by Cbh1 is less severely affected by the presence of glucose than CelD. The fact that cellobiose does not differentially affect both enzymes, but glucose does, may be a consequence of the structural differences of the catalytic channel.

### Cbh1 and CelD combine efforts to hydrolyze the same substrate molecules

**Figure 5:**
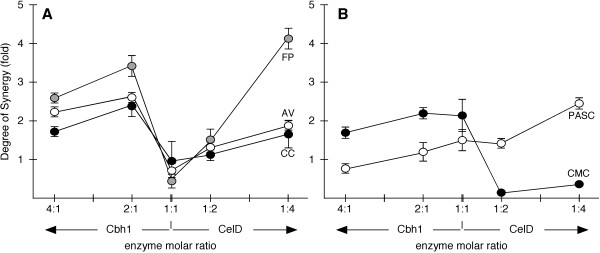
**Molar mixtures of Cbh1 and CelD, result in a combinatorial effort to attack crystalline substrates**. Panel **A** crystalline forms of cellulose, Avicel (AV), cotton cellulose (CC), filter paper (FP) and Panel **B,** partially hydrated phosphoric acid swollen Avicel (PASC) and soluble carboxymethylcellulose (CMC). 2X and 4X molar excess of Cbh1 and CelD were incubated with various forms of cellulosic substrates and the degree of synergy (ab/a + b) determined [20]. Equimolar Cbh1/CelD mixtures had no combinatorial effect (DS ≤1) on cellulose breakdown. Excess of Cbh1 over CelD resulted in a combinatorial effort (up to 345%) to attack crystalline (**A**) forms of cellulose while less crystalline forms remained unchanged (**B**). Excess of CelD over Cbh1 resulted in little or no gain of synergy (DS up to 1.91) for crystalline (**A**) substrates (except 1:4 FP) and activity was inhibited with less crystalline (**B**) substrates. For details on activity and degree of synergy, see Additional file 3: Figure S 2 and Methods.

Figure 5 shows that Cbh1/CelD combinations incubated with various cellulosic substrates develop strong synergy only under selective conditions. Cbh1 needs to be added to molar excess of at least 2 fold and the substrate has to be a crystalline form of cellulose, while excess of CelD induces little or no gain in cellulose breakdown.

Cellobiohydrolase activity (U) was determined by mixing 20 nM (2:1) and 40 nM (4:1) of Cbh1 with 10 nM of CelD or 20 nM (1:2) and 40 nM (1:4) of CelD with 10 nM of Cbh1, combined with various forms of crystalline (Figure 5A) and partially hydrated (Figure 5B) cellulosic substrates and the degree of synergy (DS = ab/a + b) was determined [20].

Equimolar Cbh1/CelD mixtures had no combinatorial effect (DS ≤1) on cellulose breakdown. Excess of Cbh1 over CelD resulted in a combinatorial effort (up to 345%) to attack crystalline (Figure 5A) forms of cellulose while less crystalline forms remained unchanged (Figure 5B). Excess of CelD over Cbh1 resulted in little or no gain in synergy (DS up to 1.91) for crystalline substrates (except 1:4 FP) and activity was inhibited with less crystalline substrates. For details on activity and degree of synergy, see Additional file 3: Figure S 2 and Methods.

Thus, molar excess of Cbh1 improved the ability to hydrolyze cellulose chains while excess of CelD only partially improved the activity, which could be correlated to the crystalinity (or available loose cellulose chain ends) of the substrate. When the substrate was PASC, an Avicel artificially swollen with phosphoric acid, there was no apparent combinatorial effort because of the excess number of untied cellulose chains. When the substrate was CMC, a soluble but substituted form of cellulose, there was no consequence when Cbh1 was present in excess or equimolar conditions, however activity was severely affected by the excess of CelD. Interestingly, when incubated alone, Cbh1 was far more active in CMC than was CelD (Figure 1A).

## Discussion

It is a widespread feature of sequenced fungal genomes to contain multiple loci that encode similar plant cell wall degrading enzymes. In *Aspergillii*, cellobiohydrolase genes are one such example. In *A. fumigatus* (or the closely related *A. niveus*), one GH6 cellobiohydrolase with a CBD and two GH7 cellobiohydrolases, one with and one without a CBD are present. Here we investigated the functionality of the two GH7 cellobiohydrolases and focus on whether the CBD domain is an essential domain for typical cellobiohydrolase function.

Fungi growing in the presence of cellulose (Avicel or cotton cellulose) expressed and secreted both enzymes to the extracellular medium. Thus, we transferred both genes *cbh1* and *celD* to a controlled high-yield expression/secretion system to recover enriched and purified Cbh1 and CelD enzyme preparations useful to study their biochemical properties.

Initially we investigated enzyme thermal tolerance (Figure 3). Both enzymes were thermolabile at temperatures of 60°C and above. However Cbh1 appeared to be significantly more thermostable at lower temperatures, 40 and 50°C compared to CelD, thus indicating that the presence of a CBD provided some thermo-protection [49,50]. We then investigated the inhibition effect of cellobiose and glucose on both enzymes (Figure 4). Both enzymes were severely inhibited by cellobiose, however Cbh1 was much less sensitive to the presence of glucose (Figure 4B). It remains unclear how the presence of a CBM could affect the inhibition by glucose. However, the opened catalytic channel in the CelD catalytic channel, could explain the high sensitivity towards the presence of glucose. Interestingly, Bu and cols [51] while probing absolute binding free energies for cellobiose and glucose on *T. reesei* GH7-cellobiohydrolase show that glucose is less stable in the catalytic channel.

We compared specific activity and other enzyme kinetic parameters using crystalline cellulosic substrates and the artificial substrate, *p*-nitrophenyl cellobiose (Table 2 and Additional file 3: Figure S 2). Cbh1 specific activity was between 16 and 4 fold more active on Avicel and cotton cellulose respectively, while CelD and Cbh1 exhibited identical specific activities when assayed with *p*NPC (Table 2). Nearly indistinguishable specific activities with *p*NPC highlights the fact that both enzymes have almost identical catalytic domains and the differences in activity on crystalline substrates emphasizes involvement of other structural binding features such as the CBD in Cbh1 and a lid-type open-loop structure in CelD.

Cbh1 k_cat_ substrate turnover rates (Table 2) were 8.47 fold higher than CelD in Avicel but only 6.05 fold higher in cotton cellulose indicating that crystalinity of the substrate had a direct effect on catalytic efficiency (k_cat_/k_m_), 1.22 versus 0.06 with Avicel and 0.98 versus 0.18 with cotton cellulose. The difference in substrate turnover rates was likely related to the presence of a CBD in Cbh1, which allowed the untying of cellulose chains from the original hydrogen bonded nanofiber while the lid-like open-loop feature on the CelD catalytic channel allowed binding and catalysis of already untied cellulose chains (Figure 1).

Thus, it seemed reasonable to assume that the amount of enzyme in the presence of a constant amount of crystalline substrate should have a synergistic effect for the protein that was capable of binding to the substrate. Indeed that was precisely what Figure 5A showed, where increasing molar amounts of Cbh1 favors specific activity over CelD, which performs at a lesser level. Therefore, protein concentration as well as substrate crystalinity differentially affects Cbh1 and CelD activity. Moreover, this could suggest some sort of interaction at the one-on-one substrate molecule level. Kurasin and Väljamäe measured Cel7A processivity and found cellulose hydrolysis was more than an order of magnitude lower than the values of the ratio of catalytic and dissociation rate constants, suggesting that the length of the obstacle-free path available for a processive run on a cellulose chain limits the processivity of cellobiohydrolase [52]. Igarashi and cols found that the sliding velocity of Cel7A on crystalline cellulose was 3.5 nm/s, and interestingly, the catalytic domain without a cellulose-binding domain moved at similar rates to that of the intact enzyme [53]. Moreover, Cel7A molecules slide along the crystalline cellulose surface and at a given point undergo collective halting [54,55].

In our experiment, when both enzymes were mixed at equal molecular amounts, Cbh1 and CelD probably occupied all untied cellulose chains and overall activity was reduced because of the poor performance of CelD on cellulose chains that were not loose. Excess of Cbh1 molecules rescued activity, because many Cbh1 molecules initiated fresh untying cellulosic fibers, halted and changed to a new strand allowing CelD to initiate a processive run. Thus, when both enzymes are mixed together they progress into a combinatorial effort whereas Cbh1 unties substrates chains and CelD hydrolyzes these cellulosic chains. When CelD was present in excess, the effect could only be partially overcome while CelD could not initiate new loose cellulosic chains only used the ones that were already available. Hence, the recovery of excessive CelD was dependent on the crystalinity of the substrate, less pronounced on CC than AV and FP showing little to no combinatorial effort effect with PASC, an artificially swollen Avicel. Activity of cellobiohydrolases on soluble (CMC) and partially hydrated substrates has been reported [56].

## Conclusions

The two cellobiohydrolases investigated in this study are similar in amino acid sequence differing mainly by the presence of a cellulose-binding domain in Cbh1 which makes this enzyme a substrate bound and CelD a soluble substrate unbound enzyme. Both enzymes have similar catalytic properties however differ in thermostability, inhibition by glucose and protein concentration dependent specific activity. The fact that Cbh1 binds to its substrate and specific activity is dependent on protein concentration suggests that both enzymes employ a combinatorial effort in attacking the crystalline forms of cellulose.

## Methods

### Materials

Cellulosic and hemicellulosic substrates were purchased from the best source possible, Sigma Aldrich, MO and Megazyme, UK. For synthesis of APTS-labeled cellopentaose 1 mg of cellopentaose, β-D-Glc-[1 → 4])_4_-D-Glc, D(+)-cellopentaose (Sigma Aldrich, MO) was mixed with 10 μl of 10 mg APTS (8-aminopyrene-1,3,6-trisulfonic acid trisodium salt) in 200 μl of 25% acetic acid and 10 μl of 1 M sodium cyanoborohydride in DMSO, heated at 80°C for 60 min and purified as described in [57]. The cellulosic substrates used throughout were carboxymethylcellulose (CMC), cotton linters, SigmaCel50 (CC) crystalinity index (CI) of 91.2, Avicel PH-101 91.7 CI as determined by the x-ray diffraction method [58], phosphoric acid swollen-Avicel (PASC) and filter paper, Whatman #3 (FP). Proteins were quantified by the Bradford method [59], validated for purity by SDS-PAGE [60] and used for biochemical studies.

Standard *A. nidulans* minimal medium (MM) and general cultivation techniques were used throughout this work and were based on [61,62].

#### Construction of pEXPYR-client protein plasmids

The pEXPYR *Aspergillus* “shuttle” expression plasmid for expression and secretion of client proteins was used [46]. PCR-amplified gene-fragments (for primers of gene models see Additional file 3: Figure S 2) were digested with NotI and XbaI, isolated by gel excision of a thin-slice from a 0.8% agarose electrophoresis gel, purified with QIAquick Gel Extraction kit (Quiagen), ligated onto NotI/XbaI digested pEXPYR plasmid with T4-fast ligase (Promega, WI) and transformed into Ca^+^ competent *Escherichia coli* TOP 10 F’ competent cells (Invitrogen, CA). Random ampicillin-resistant colonies were selected and grown in 5 ml LB-ampicillin broth, plasmids purified [63], restricted with NotI/XbaI and insert size verified by 1% agarose gel electrophoresis [63]. Plasmids with the correct insert size DNA were fully sequenced at the Oklahoma State University Core Facility and clones with the correct DNA sequence used for transformation. Recombinant pEXPYR-Cbh1 or pEXPYR-CelD plasmid was introduced through integrative transformation into the *A. nidulans* strain FGSC A773 (*pyrGpyroA*) genome [64] and recombinants selected on MM supplemented with 1 mM pyridoxine and 100 μg/ml of zeocin. Five *pyrG*^*+*^, zeocin resistant transformants were grown on 10 ml MM, pyridoxine and 5% maltose containing plates for 48 h at 37°C. Accumulation of Cbh1 and CelD in the medium was analyzed by SDS-PAGE and one transformant for each enzyme was used for further investigation.

#### Production and secretion of client proteins

10^7^-10^8^ spores/ml were inoculated in liquid minimal medium supplemented with 0.5 to 15% of maltose, distributed onto dishes (20 ml in 150 mm Petri-dishes and 500 ml onto cafeteria trays) and incubated (stationary) at 37°C for 2–3 days. The mycelial mat was lifted with a spatula and discarded and the medium collected by filtration, centrifuged at 10,000*x*g for 10 min prior to concentration by ultra-filtration (10,000 kDa cutoff Amicon), quantified by the Bradford method [59], validated for purity by SDS-PAGE [60] and used for biochemical studies.

#### Cbh1/CelD purification

For biochemical studies, the recombinant Cbh1 and CelD proteins were produced by heavy inoculation of fresh conidia onto Petri dishes containing MM supplemented with pyridoxine and 5% maltose and incubated at 37°C for 48 h. Cbh1 and CelD were routinely recovered with this stationary incubation method and proteolysis avoided due to the 2 day incubation period. The medium was harvested by filtration, centrifuged at 10,000*xg* and concentrated by ultra filtration on Amicon 10 kDa cut-off micro columns. The majority of the protein content recovered from culture filtrates was Cbh1 or CelD.

Crude ultra filtrated protein extracts were resolved by SDS-PAGE, Comassie-blue stained (CB) and the SDS removed by successive washes with 25% isopropanol solution. After transferred to 50 mM ammonium acetate buffer pH 5.0 a 1% carboxymethylcellulose (CMC) solution was infused into the SDS-free polyacrylamide gel, incubated at 37°C for 120 min, and stained with Congo red [65-68].

Cbh1 and CelD were purified by two steps. The concentrated and dialyzed protein samples (500 μl aliquots) were applied to ion exchange Resource Q® column equilibrated with 20 mM sodium phosphate buffer, pH 7.4 and proteins eluted with a linear 0 to 1 M sodium chloride gradient (Äkta Purifier, GE). Fractions active on *p*NPC were collected and loaded onto a Superdex G-75® (10x30 mm) gel filtration column, equilibrated with 50 mM ammonium acetate buffer, pH 5.0 and eluted fractions showing enzymatic activity were analyzed by SDS-PAGE. Single band containing fractions were combined, concentrated and used for further biochemical analysis. The flow rate used for both chromatographic steps was 0.5 ml min^-1^. Purified Cbh1 and CelD fractions were validated by SDS-PAGE (Additional file 1: Table S 1), and *p*NPC, *p*NPG activity measurement comparisons (Additional file 3: Figure S 2).

#### Optimal pH, temperature and thermostability

Optimal pH was measured at 50°C in the presence of 18 mM *p*NPC with the pH ranging from pH 3.0 to pH 8.0 using 50 mM phosphate/citrate buffer. Optimal enzyme operating temperatures for Cbh1 and CelD were measured at their optimal pH, 5.0, with temperatures ranging from 30°C to 80°C. Thermal stability of Cbh1 and CelD was tested at optimal pH and exposure for various times (up to one hour). Purified enzyme in 50 mM ammonium acetate (without substrate) was incubated at temperatures ranging from 40 to 70°C. Samples were drawn from a master mix and residual activity assayed with 18 mM *p*NPC.

#### Cellulose specific CelD or Cbh1, CBD-dependent binding

To reveal the functionality and specificity of the predicted CBM1 domain, the binding of Cbh1 or CelD was evaluated by a pull-down assay. 20 μg of Cbh1 or CelD was incubated at 4 C for 30 min in a rotary shaker, with 200 μl of 30% cotton cellulose or Avicel slurry in 50 mM, pH 5.0, ammonium acetate buffer. The reaction was centrifuged at 14,000*x*g for 15 min, supernatant (free fraction) collected and concentrated in an Amicon, 10 kDa cutoff ultra filtration column mixed with 2X Laemmli buffer and subjected to SDS-PAGE. The bound fraction was released from the Avicel or cotton linter or Avicel slurry by addition of 40 μl of 2X Laemmli buffer, vigorous agitation and boiling for 10 min. The SDS protein-solubilized slurry was centrifuged and supernatant (bound fraction) subjected to SDS-PAGE.

#### Cellobiohydrolase activities, substrate- and protein-dependent kinetics

Substrate specificity of cellobiohydrolases was determined by incubating 1 μg of Cbh1 or CelD with a, 1% slurry of cotton cellulose (Sigmacell 50), Avicel PH-101, PASC or 1% solution of CMC or 18 mM *p*NPCellobiose, incubated for 120 min or as indicated at 50°C and the release of reducing sugars determined with the DNS method [69]. Specific activity was defined as U per mg protein at 50°C whereas U was the amount of enzyme that produced 1 μmole of reducing sugar (glucose or cellobiose) per minute. Activity towards starch, polygalacturonic acid, wheat arabinoxylan, arabinan from sugar beet, xylan birchwood, xylan beechwood and xyloglucan from tamarind could not be detected and is not shown.

Michaelis-Menten kinetic constants were determined from Lineweaver–Burk plots. Reaction rates were measured using Avicel and cotton cellulose ranging from 0 to 100 mg/ml of insoluble substrate suspended in 50 mM ammonium acetate buffer pH 5.0. Reactions were carried out over a 120-min period at 50°C, boiled, and the released reducing sugars determined by DNS assay [69]. The raw substrate activity plots are shown in Additional file 4: Figure S 3.

#### Homology molecular modeling

The atomic coordinates of the cellobiohydrolase I (CBH IB) from *Talaromyces emersonii* (PDBID: 1Q9H, [47]) was used as template for generating structural models of both CBHI and CelD by restraint-based modeling as implemented in the program MODELLER [71]. To guarantee sufficient conformational sampling, an ensemble of 50 models was built, from which the best final model was selected based on evaluation of stereo chemical values from MOLPROBITY [72], the objective function from MODELLER (DOPE score) and by visual inspection. Those models were then minimized using the steepest descent minimization algorithm as implemented in the UCSF chimera software [73]. Incomplete side-chains were replaced using the Dunbrack rotamer library [74].

#### Cellobiose and glucose Inhibition

To analyze Cbh1 and CelD inhibition, 1 μg enzyme was incubated in 18 mM *p*NPC dissolved in 50 mM ammonium acetate buffer pH 5.0 with glucose or cellobiose added in a range from 0 to 100 mM at 50°C. After 15 min, enzyme activity was stopped by adding 100 μl of a 2% Na_2_CO_3_. The *p*NPC chromophore release was spectrophotometrically quantified at 410 nm with a Multimode Infinte M200 Reader (Tecan, SC).

## Abbreviations

CaZy, Carbohydrate-Active enZYmes Database (http://www.cazy.org/); Cbh1, CaZy GH7 (Cel7A) cellobiohydrolase 1; CelD, CaZy GH7 (Cel7B) cellobiohydrolase D; CI, Crystalinity index; CD, Catalytic domain; CBD, Cellulose-binding domain; CMC, Carboxymethylcellulose; AV, Avicel; CC, Cotton cellulose; DNS, Dintrosalicilic acid; DS, Degree of synergism; FP, Whatman #3 filter paper; MM, Minimal Aspergillus medium; pNPC, Para-nitrophenylcellobiose; pNPG, Para-nitophenylglucose; PASC, Phosphoric acid swollen (Avicel) cellulose; SDS-PAGE, Sodium dodecyl sulfate polyacrylamide gel electrophoresis.

## Competing interests

The authors are not aware of any affiliations, memberships, funding, or financial holdings that might be perceived as affecting the objectivity of this article.

## Authors’ contributions

Conceived and designed the experiments: RAP, MLTMP and AM. Made the 3D-structural modeling: MTM and FMS. Performed the experiments: ARLD, FS and TAG and contributed equally to this work. Analyzed the data: FS, ARLD, TAG, RAP and MTM. Wrote the paper: RAP, ARLD, FS, FMS, MLTMP and MTM. All authors read and approved the final manuscript.

## Supplementary Material

Additional file 1 **Table S1.** Primers used in this study. **Table S2.** Cbh1 and CelD substrate binding competition. **Table S3.** Cbh1 and CelD *p*NPC and *p*NPG activity . Click here for file

Additional file 2 **Figure S1.** Time course *A. nidulans* client expression and secretion of *A. niveus* Cbh1 (A) and CelD (B) and purified enzymes (C). Note that after the second day native Cbh1 and CelD are subjected to proteolytic degradation in the medium. Click here for file

Additional file 3 **Figure S2.**Cellobiohydrolase substrate dependent kinetics with crystalline cellulosic fibers. Michaelis-Menten substrate dependent, avicel (open symbols) and cotton linters (closed) cellobiohydrolase activity of Cbh1 (A) and CelD (B). Nearly equal amounts of enzyme (9.3 nM Cbh1 and 10.7 nM CelD) were incubated with increasing amounts of substrate, avicel or cotton linters and specific velocity (μmol/min) determined after a 120 min reaction period at 40 C. Click here for file

Additional file 4 **Figure S3.** Cbh1 and CelD differential thermal inactivation . Click here for file
