# Supplementary material for: Two structurally discrete GH7-cellobiohydrolases compete for the same cellulosic substrate fiber
Source: Biotechnol Biofuels. 2012 Apr 11;5:21. doi: 10.1186/1754-6834-5-21 (PMC3431977; doi:10.1186/1754-6834-5-21)
Supplement: Additional file 1 — Table S1. Primers used in this study. Table S2. Cbh1 and CelD substrate binding competition. Table S3. Cbh1 and CelD pNPC and pNPG activity . [file 1754-6834-5-21-S1.doc]

**Supplemental Tables and Figures**

**Table S1** Primers used in this study

Cbh1 fwd - NNNGCGGCCGCCAGCAGGTCGGTACTTCCCAGGCGGAAGTG

Cbh1 rev - NNNTCTAGAGGCTAATTACTACAGGCACTGAGAGTAATAATCATTCAG

CelD fwd - NNNGCGGCCGCCAGCAGGTCGGCACTCAGACACCGGAAACC

CelD rev - NNNTCTAGAGGCTAATTATTAACCTTCGTAGGTCGAACCAATGGGACC

**Table S3** Cbh1 and CelD *p*NPC and *p*NPG activity

__________Specific activity_________ %GL FOLD

E *p*NPC *p*NPG

U/mg.prot U/mg.prot

Cbh1 26.198 ± 0.195 0.551 ± 0.635 2.1% 48

CelD 27.306 ± 1.387 0.471 ± 0.754 1.7% 58

**Figure S1** Time course *A. nidulans* client expression and secretion of *A. niveus* Cbh1 (**A**) and CelD (**B**) and purified enzymes (**C**). Note that after the second day native Cbh1 and CelD are subjected to proteolytic degradation in the medium.

**Figure S2**. Cellobiohydrolase substrate dependent kinetics with crystalline cellulosic fibers.

Michaelis-Menten substrate dependent, avicel (open symbols) and cotton linters (closed) cellobiohydrolase activity of Cbh1 (**A**) and CelD (**B**). Nearly equal amounts of enzyme (9.3 nM Cbh1 and 10.7 nM CelD) were incubated with increasing amounts of substrate, avicel or cotton linters and specific velocity (μmol/min) determined after a 120 minutes reaction period at 40^0^C.

**Fig S3** Cbh1 and CelD differential thermal inactivation
