# Supplementary figures and images for: Two structurally discrete GH7-cellobiohydrolases compete for the same cellulosic substrate fiber
Source: Biotechnol Biofuels. 2012 Apr 11;5:21. doi: 10.1186/1754-6834-5-21 (PMC3431977; doi:10.1186/1754-6834-5-21)

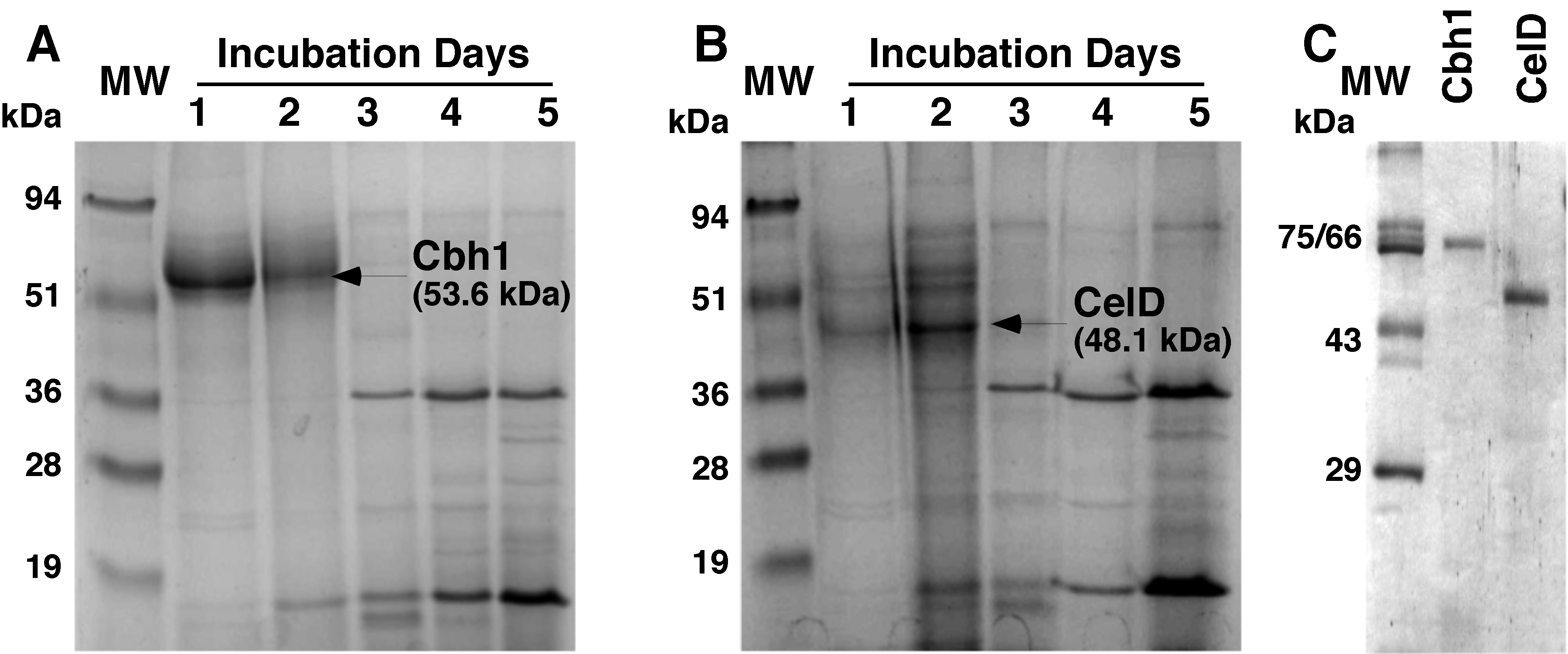

Supplement: Additional file 2 — Figure S1. Time course A. nidulans client expression and secretion of A. niveus Cbh1 (A) and CelD (B) and purified enzymes (C). Note that after the second day native Cbh1 and CelD are subjected to proteolytic degradation in the medium. [file 1754-6834-5-21-S2.tiff]

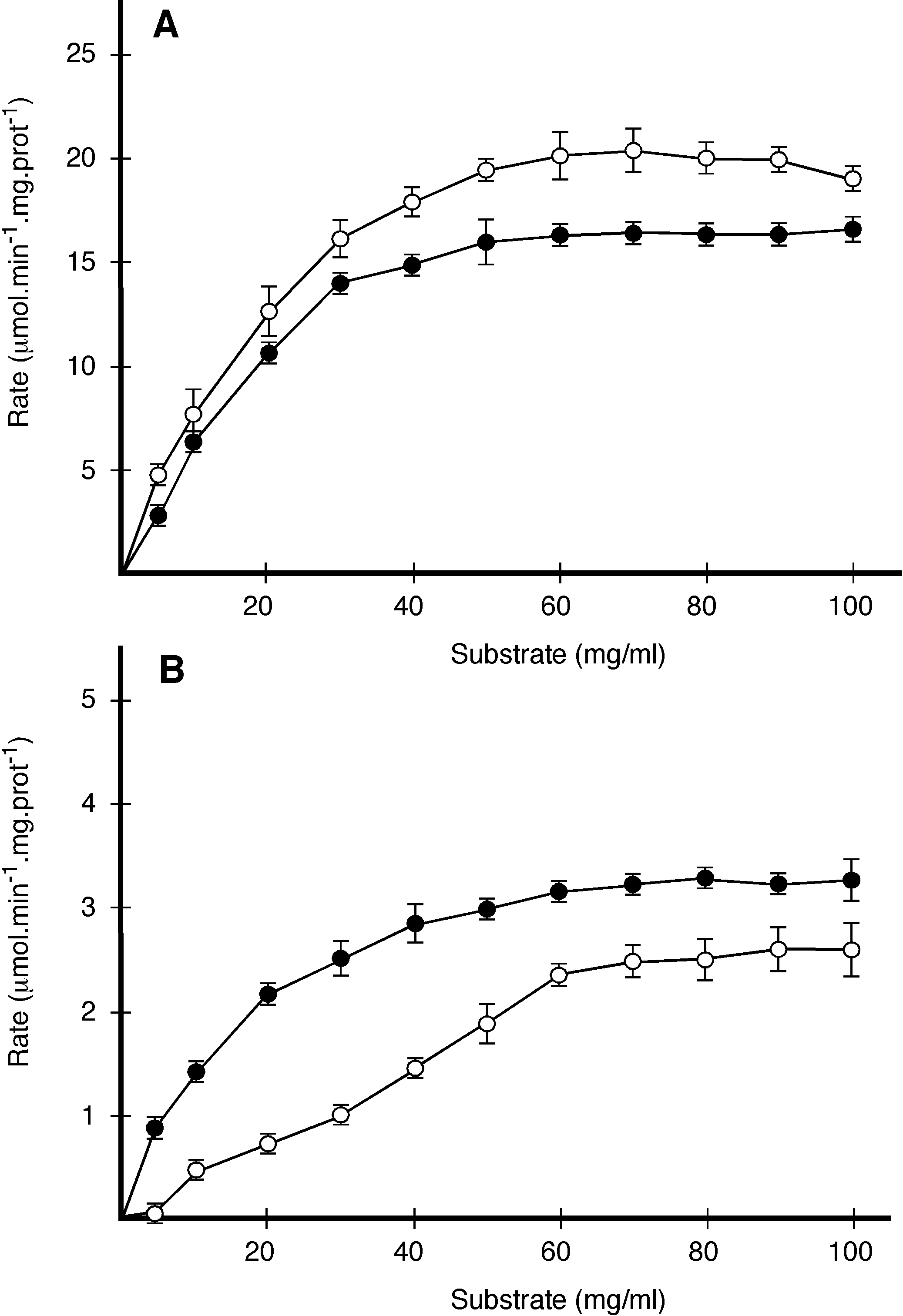

Supplement: Additional file 3 — Figure S2.Cellobiohydrolase substrate dependent kinetics with crystalline cellulosic fibers. Michaelis-Menten substrate dependent, avicel (open symbols) and cotton linters (closed) cellobiohydrolase activity of Cbh1 (A) and CelD (B). Nearly equal amounts of enzyme (9.3 nM Cbh1 and 10.7 nM CelD) were incubated with increasing amounts of substrate, avicel or cotton linters and specific velocity (μmol/min) determined after a 120 min reaction period at 40 C. [file 1754-6834-5-21-S3.tiff]

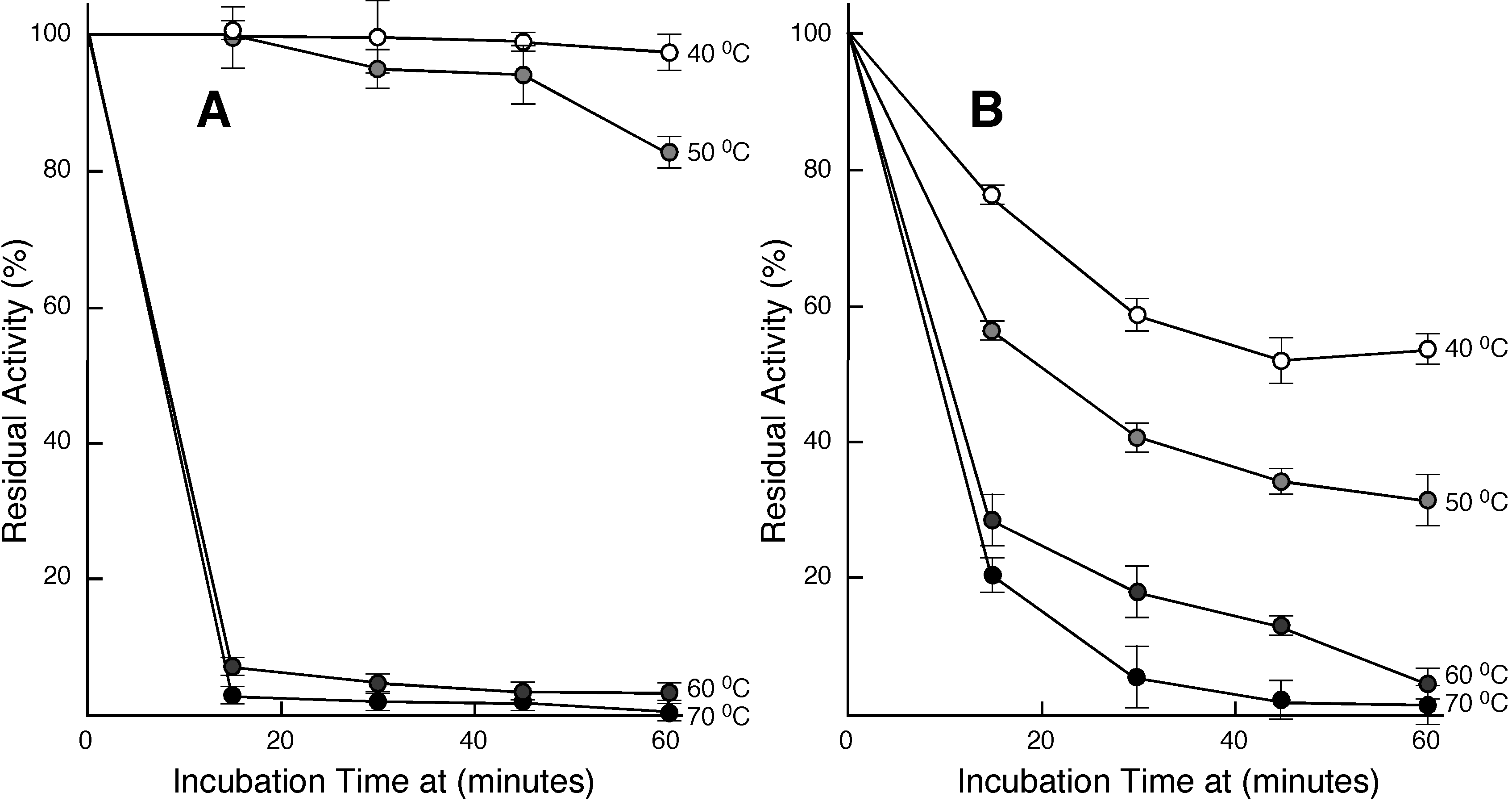

Supplement: Additional file 4 — Figure S3. Cbh1 and CelD differential thermal inactivation . [file 1754-6834-5-21-S4.tiff]
